# Band-ligation followed by anchor pronged clips for greater post-endoscopic submucosal dissection defect closure

**DOI:** 10.1055/a-2729-2052

**Published:** 2025-12-03

**Authors:** Kaho Nakatani, Noriko Nishiyama, Kazuhiro Kozuka, Yukiko Koyama, Takanori Matsui, Tatsuo Yachida, Hideki Kobara

**Affiliations:** 1 38078 Department of Gastroenterology and Neurology, Faculty of Medicine, Kagawa University, Kita, Japan

The efficacy of defect closure following gastric endoscopic submucosal dissection (ESD) in reducing delayed bleeding has been recently reported 1 2 3 . However, due to the specific thickness of the gastric wall, achieving secure and reliable closure of post-ESD defects remains technically challenging. In particular, grater defect closure is bothersome. We herein report the successful closure of grater defects using a novel technique which combines our developed endoscopic ligation with O-ring closure (E-LOC; Fig. 1 **a** 4 ), and anchor pronged clips (MANTIS clips; Boston Scientific, Marlborough, MA) applied in a hold-and-drag manner ( Fig. 1 **b** 5 ), called as M-LOC. E-LOC closure has a significant strength of preventing submucosal dead space creation after closure by anchoring a muscular layer 4 .

**Fig. 1:**
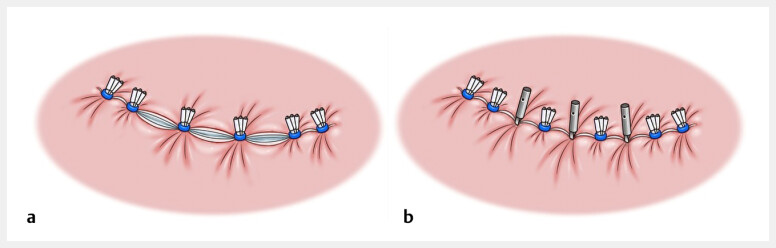
**a** E-LOC enabled defect approximation without dead space. **b** MANTIS clips provided secure and sustained closure. Source: Davinch Medical Illustration Office, Tokyo, Japan. E-LOC, endoscopic ligation with O-ring closure.

An 89-year-old man, taking aspirin, who presented with a 55-mm flat-elevated early gastric carcinoma located in the lesser curvature at the angle underwent ESD, resulting in a 75-mm mucosal defect ( Fig. 2 **a** ). After informed consent was obtained, the defect was closed with the M-LOC technique ( Video 1 ). E-LOC was first applied to approximate the defect ( Fig. 2 **b** ), followed by additional placement of MANTIS clips to fill residual gaps and reinforce closure. Complete closure was achieved in 36 minutes, and the sustained closure on postoperative day (POD) 3 was endoscopically confirmed ( Fig. 2 **c** and **d** ).

**Fig. 2:**
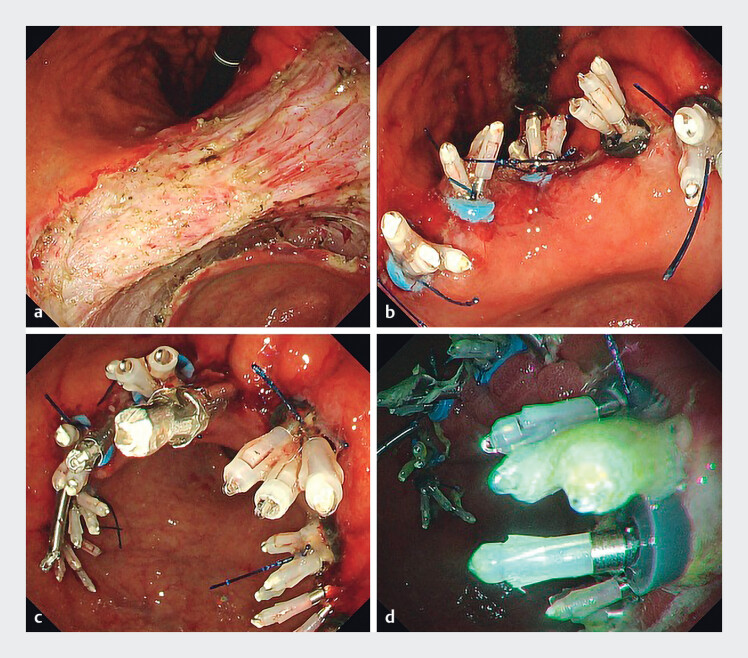
Details of the closure in an 89-year-old patient. **a** After standard ESD, a 75-mm-diameter defect remained. **b** E-LOC was performed first. **c** MANTIS clips were added to achieve complete closure. **d** The closure was sustained at postoperative day (POD) 3. ESD, endoscopic submucosal dissection; E-LOC, endoscopic ligation with O-ring closure.

Video showing the procedure for closing a greater post-ESD defect using the M-LOC technique. Source for graphical illustrations: Davinch Medical Illustration Office, Tokyo, Japan. ESD, endoscopic submucosal dissection.Video 1

In the second case, an 80-year-old man, taking a direct oral anticoagulant, underwent ESD for a 50-mm flat type of early gastric carcinoma on the lower curvature of the gastric body, resulting in a 75-mm defect ( Fig. 3 **a** ). M-LOC achieved complete closure in 45 minutes and maintained it on POD 3 ( Fig. 3 **b** and **c** ). No delayed complications occurred in either case. With this concept of closure, E-LOC enabled defect approximation without dead space, followed by the application of MANTIS clips for maintaining reinforce and sustain closure.

**Fig. 3:**
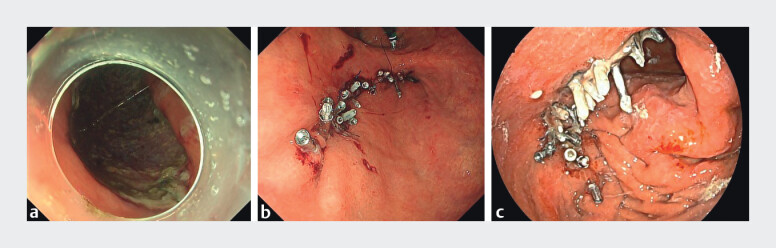
Details of the closure in an 80-year-old patient. **a** After standard ESD, a 75-mm-diameter defect remained. **b** Complete closure was achieved. **c** The closure was sustained at POD 3. ESD, endoscopic submucosal dissection; POD, postoperative day.

The M-LOC technique may be an effective option for closing greater post-ESD gastric defects, leading to decreased delayed complications.

Endoscopy_UCTN_Code_TTT_1AO_2AZ

## Notes

Endoscopy E-Videos https://eref.thieme.de/e-videos
*E-Videos* is an open access online section of the journal *Endoscopy* , reporting on interesting cases and new techniques in gastroenterological endoscopy. All papers include a high-quality video and are published with a Creative Commons CC-BY license. Endoscopy E-Videos qualify for HINARI discounts and waivers and eligibility is automatically checked during the submission process. We grant 100% waivers to articles whose corresponding authors are based in Group A countries and 50% waivers to those who are based in Group B countries as classified by Research4Life (see: https://www.research4life.org/access/eligibility/ ). This section has its own submission website at https://mc.manuscriptcentral.com/e-videos .
